# AMPK β1 reduces tumor progression and improves survival in p53 null mice

**DOI:** 10.1002/1878-0261.12079

**Published:** 2017-06-28

**Authors:** Vanessa P. Houde, Sara Donzelli, Andrea Sacconi, Sandra Galic, Joanne A. Hammill, Jonathan L. Bramson, Robert A. Foster, Theodoros Tsakiridis, Bruce E. Kemp, Giuseppe Grasso, Giovanni Blandino, Paola Muti, Gregory R. Steinberg

**Affiliations:** ^1^ Department of Oncology McMaster University Hamilton Canada; ^2^ Department of Medicine McMaster University Hamilton Canada; ^3^ Oncogenomic and Epigenetic Unit Italian National Cancer Institute ‘Regina Elena’ Rome Italy; ^4^ St. Vincent's Institute of Medical Research and Department of Medicine University of Melbourne Australia; ^5^ Department of Pathology and Molecular Medicine McMaster University Hamilton Canada; ^6^ Department of Pathobiology Ontario Veterinary College University of Guelph Canada; ^7^ Mary MacKillop Institute for Health Research Australian Catholic University Fitzroy Australia; ^8^ Department of Biochemistry and Biomedical Sciences McMaster University Hamilton Canada

**Keywords:** ACC, metabolism, cancer, lipogenesis

## Abstract

The AMP‐activated protein kinase (AMPK) is a heterotrimeric protein complex that is an important sensor of cellular energy status. Reduced expression of the AMPK β1 isoform has been linked to reduced survival in different cancers, but whether this accelerates tumor progression and the potential mechanism mediating these effects are not known. Furthermore, it is unknown whether AMPK β1 is implicated in tumorigenesis, and if so, what tissues may be most sensitive. In the current study, we find that in the absence of the tumor suppressor p53, germline genetic deletion of AMPK β1 accelerates the appearance of a T‐cell lymphoma that reduces lifespan compared to p53 deficiency alone. This increased tumorigenesis is linked to increases in interleukin‐1β (IL1β), reductions in acetyl‐CoA carboxylase (ACC) phosphorylation, and elevated lipogenesis. Collectively, these data indicate that reductions in the AMPK β1 subunit accelerate the development of T‐cell lymphoma, suggesting that therapies targeting this AMPK subunit or inhibiting lipogenesis may be effective for limiting the proliferation of p53‐mutant tumors.

**Abbreviations**
ATG13: autophagy‐related protein 13
BSA: bovine serum albumin
CD3: cluster of differentiation 3
DMEM: Dulbecco's modified Eagle's medium
FBS: fetal bovine serum
FoxP3: forkhead box P3
HIF1α: hypoxia‐inducible factor 1‐alpha
LC3B: microtubule‐associated proteins 1A/1B light chain
LDL: low‐density lipoprotein
LPS: lipopolysaccharides
mTOR: mechanistic target of rapamycin
p53: tumor protein p53
p62: sequestosome 1
PBS: phosphate‐buffered saline
ROS: reactive oxygen species
S6K: ribosomal protein S6 kinase
S6: ribosomal protein S6
Ser: serine
Thr: threonine
TNF: tumor necrosis factor
ULK: UNC‐51‐like kinase
WT: wild‐type

## Introduction

While it is now established that obesity and diabetes can increase the risk of developing many types of cancer, the molecular signals that collaborate with oncogenes to accelerate tumorigenesis under conditions of nutrient excess are still not fully understood. The AMP‐activated protein kinase (AMPK) is an α, β, γ heterotrimer that senses changes in cellular energy status, and whose activity is reduced under conditions of nutrient surplus such as obesity and diabetes (Ruderman *et al*., 2013; Steinberg and Kemp, 2009). AMPK activation by the upstream AMPK kinase LKB1 causes regulation of multiple branches of cellular metabolism that may be important for limiting cancer cell proliferation (Mihaylova and Shaw, 2011). This involves the inhibition of energy‐consuming processes such as fatty acid and protein biosynthesis, while increasing catabolism by promoting autophagy and fatty acid oxidation (Carling *et al*., 2012; Faubert *et al*., 2015; Mihaylova and Shaw, 2011). In addition to direct regulation of key metabolic checkpoints, AMPK may also inhibit tumorigenesis through upregulation of transcription factors such as the tumor suppressor p53, which is mutated in approximately 80% of all cancers (He *et al*., 2014; Jones *et al*., 2005; Lee *et al*., 2012). Mutation or genetic deficiency of p53 may also act to suppress AMPK activity (Zhou *et al*., 2014), suggesting that the activities of p53 and AMPK may be intimately linked to match energy availability with cell proliferation (Adamovich *et al*., 2014). While many studies have examined the interaction between AMPK and the tumor suppressor p53 in cultured cells, the importance of this pathway to tumorigenesis has not been studied *in vivo*.

Increases in fatty acid synthesis are required to sustain rapid proliferation in many types of cancer [for review, see (Hanahan and Weinberg, 2011; Hirschey *et al*., 2015)] including prostate and non‐small‐cell lung cancer (NSCLC) (Shah *et al*., 2016; Svensson *et al*., 2016; Villani *et al*., 2016). Rates of fatty acid synthesis are governed by the expression of ATP‐citrate lyase (ACLY), acetyl‐CoA carboxylase (ACC), and fatty acid synthase (FAS) (Hanahan and Weinberg, 2011; Hirschey *et al*., 2015; Shah *et al*., 2016; Svensson *et al*., 2016; Villani *et al*., 2016). While AMPK may regulate multiple branches of the lipogenic pathway, direct phosphorylation of ACC has been shown to result in the inhibition of ACC activity, thereby preventing the conversion of acetyl‐CoA to malonyl‐CoA and blocking the entire lipogenic program (Fullerton *et al*., 2013; Munday *et al*., 1988). As such, AMPK may exert antiproliferative effects through both inhibition of lipogenesis and by inhibiting cell cycle progression.

Despite the many connections between AMPK and cancer cell proliferation, mutations in the AMPK β1 subunit are not common in cancers (Forbes *et al*., 2015) and recent studies have suggested that inhibition of AMPK may make cancer cells more sensitive to apoptosis (Jeon *et al*., 2012; Shackelford *et al*., 2013; Zadra *et al*., 2015). Thus far, only one study has examined the effects of germline deletion of AMPK in a mouse model of tumorigenesis where the authors observed that deletion of the AMPK α1 isoform promoted B‐cell lymphoma when crossed to c‐Myc transgenic mice (Faubert *et al*., 2013). To the best of our knowledge, the *in vivo* importance of other AMPK subunits in combination with genetic loss of tumor‐suppressing pathways has not been studied. However, this is important given that the 12 different AMPK heterotrimer combinations have unique tissue‐specific expression, enzyme activities, and substrates (Ross *et al*., 2016) and may have differential effects in distinct settings of oncogenesis (i.e., when interacting with distinct tumor‐promoting pathways).

The AMPK β1 isoform plays a vital role in regulating AMPK heterotrimer formation and enzyme activity in mouse liver and hematological cells such as macrophages and T cells where it increases fatty acid oxidation, suppresses fatty acid synthesis, and inhibits inflammatory pathways (Blagih *et al*., 2015; Dzamko *et al*., 2010; Fullerton *et al*., 2015; Galic *et al*., 2011). It is also the primary subunit which is targeted by small‐molecule direct AMPK activators such as A769662 (Cool *et al*., 2006; Sanders *et al*., 2007; Scott *et al*., 2014), MT‐63‐78 (Zadra *et al*., 2014), and salicylate (Hawley *et al*., 2012), all of which have been shown to reduce cancer cell proliferation in some (Huang *et al*., 2008; O'Brien *et al*., 2015; Zadra *et al*., 2014), but not all studies (Vincent *et al*., 2015). Elevated AMPK β1 expression has also been linked to increased lymphoma (Hoffman *et al*., 2013) and ovarian cancer survival (Li *et al*., 2012), tumor types that are commonly associated with mutations in p53. In addition the DNA‐damaging agent etoposide increases AMPK β1 expression and overexpression of AMPK β1 reduces cellular proliferation *in vitro* (Li *et al*., 2003). However, it is not known whether AMPK β1 can inhibit tumorigenesis *in vivo* and in what tissues/tumor types this may be most important.

In the current study, we generated AMPK β1‐p53 null mice and found that this combined genotype accelerates the development of a lethal T‐cell lymphoma compared to p53 deletion alone. This increased lethality is linked with reductions in tumor ACC phosphorylation and increases in interleukin 1β and lipid synthesis. These data indicate that AMPK β1 containing complexes and AMPK activity may be important to limit tumorigenesis and improve survival in p53‐deficient T‐cell lymphomas.

## Results

### The genetic deletion of AMPK β1 accelerates the development of T‐cell lymphoma in p53‐deficient mice

To investigate the connections between AMPK and p53 *in vivo,* we crossed AMPK β1 null mice (Dzamko *et al*., 2010) to mice lacking p53 (Donehower *et al*., 1992). AMPK β1 null mice (Dzamko *et al*., 2010) were generated on a C57Bl6 background as described and p53 null mice had been backcrossed to a C57Bl6 background for at least 10 generations. Genetic deletion of p53 results in the development of a T‐cell lymphoma at approximately 6 months of age (Jacks *et al*., 1994). In contrast, AMPK β1 null mice have a normal lifespan with no signs of elevated tumorigenesis (Dzamko *et al*., 2010). The AMPK β1−/− mice were born at the expected Mendelian ratio (Fig. S1). In contrast, the homozygous p53−/− mice were born at a lower Mendelian frequency (Fig. S1). In order to generate the p53−/− AMPK β1−/− mice, we then crossed and interbred, as described in Fig. S1, the different mice genotypes.

**Figure 1:**
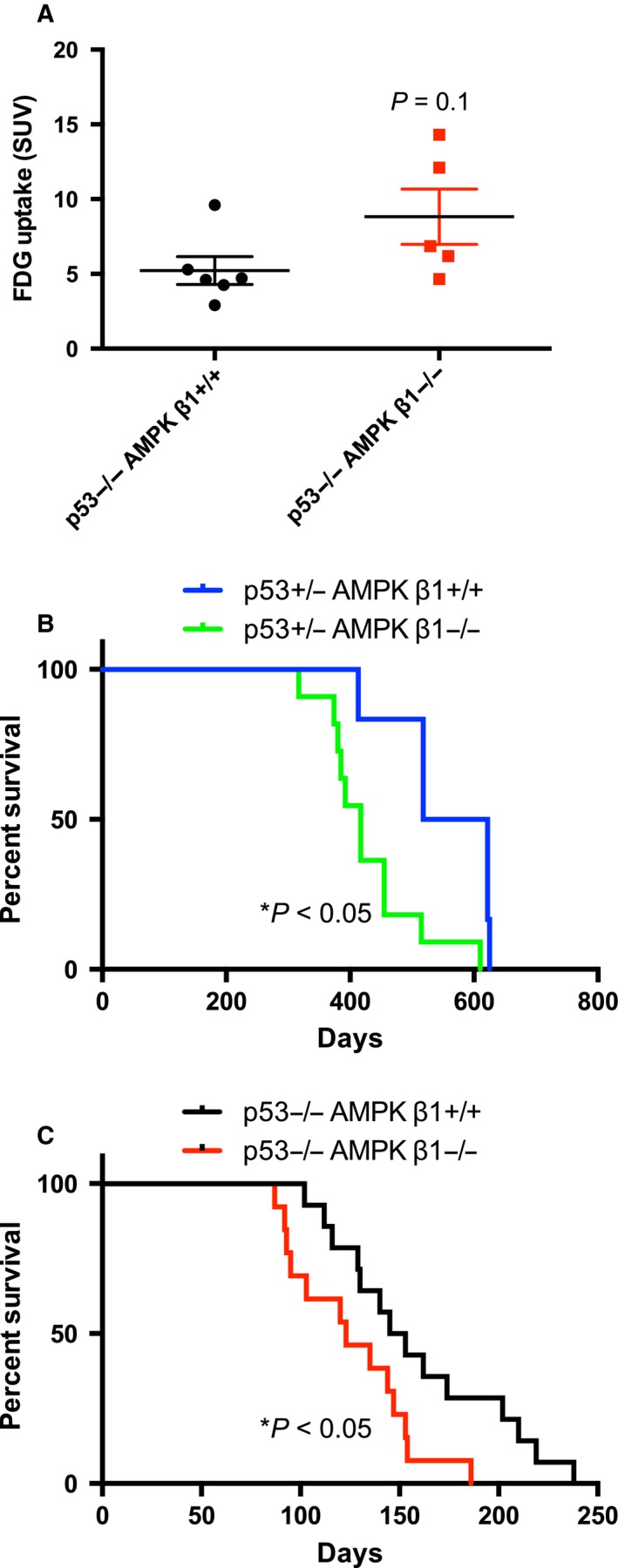
Accelerated tumor progression in the p53−/− AMPK β1−/− mice. (A) Three‐month‐old mice were submitted to CT/PET scan, and thymic ^18^F‐FDG uptake was then quantified for the p53−/− AMPK β1+/+ and p53−/− AMPK β1−/− mice. SUV, standardized uptake values. *n* = 5–6 per genotypes. Results are the means ± SEM *P < 0.05, **P < 0.01, ***P < 0.001, ****P < 0.0001 using two‐tailed Student's *t*‐test. (B) Kaplan–Meier survival curve showing survival of the p53+/− AMPK β1+/+ (blue) and p53+/− AMPK β1−/− (green) mice (*P < 0.05 by Wilcoxon test). *n* = 6–11 mice per group. C) Kaplan–Meier survival curve showing survival of the p53−/− AMPK β1+/+ (black) and p53−/− AMPK β1−/− (red) mice (*P < 0.05 by Wilcoxon test). *n* = 13–14 mice per group.

Lifespan of mice was monitored over time by animal technicians in a blinded manner. At 3 months of age, we assessed *in situ* [^18^F]fluorodeoxyglucose (FDG) uptake using positron emission tomography/computed tomography (PET/CT) imaging and found that there was a tendency for p53−/− AMPK β1−/− mice to have increases in thymic FDG uptake compared to p53−/− AMPK β1+/+ mice (Fig. 1A), suggesting earlier tumor onset in p53−/− AMPK β1−/− compared to p53−/− AMPK β1+/+ mice. Consistent with earlier tumor onset, mice heterozygous for p53 (p53+/−) or homozygous for p53 (p53−/−) in the absence of AMPK β1 had reduced survival (Fig. 1B,C). These data indicate that AMPK β1 is important for limiting tumor development and lethality following homozygous or heterozygous deletion of the tumor suppressor p53.

**Figure 2:**
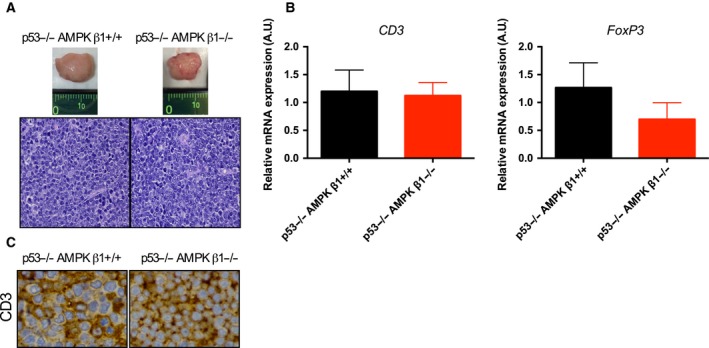
Development of a T‐cell lymphoma in the p53−/− AMPK β1+/+ and p53−/− AMPK β1−/− mice. (A) Tumors were collected from p53−/− AMPK β1+/+ and p53−/− AMPK β1−/− mice at endpoint. Pictures of tumor (top). The ruler units are millimeters. Tumor sections were stained with hematoxylin and eosin (bottom). Original magnification, ×40. (B) mRNA expression of T‐cell markers CD3 and FoxP3 in tumors of p53−/− AMPK β1+/+ and p53−/− AMPK β1−/− mice at endpoint. *n* = 6–7 mice per group (C) Immunohistochemical staining of T‐cell marker CD3 (original magnification, ×40).

At endpoint (which was earlier in the p53−/− AMPK β1−/− compared to p53−/− AMPK β1+/+ mice), pathological analysis indicated that both genotypes developed a similar tumor in the thymus (Fig. 2A). The size and weight of these tumors was not different between genotypes at endpoint (Fig. 2A). Both genotypes also developed a much smaller proportion other tumors that match the tumor spectrum observed in the p53−/− mice (Table 1) (Jacks *et al*., 1994). p53 null mice are known to develop thymic T‐cell lymphomas (Donehower *et al*., 1992; Jacks *et al*., 1994). Therefore, we next examined two markers of T cells (CD3 and FoxP3) and found that expression levels were similar in the tumors of p53−/− AMPK β1+/+ and 53−/− AMPK β1−/− mice (Fig. 2B). Confirming these findings, immunohistochemical (IHC) staining of tumor sections indicated similar expression of the T‐cell marker CD3 (Fig. 2C). Collectively, these findings suggest that genetic deletion of AMPK β1 does not change the tumor spectrum but rather accelerates tumor progression.

**Table 1:** Incidence of tumors in p53−/− AMPK β1+/+ and p53−/− AMPK β1−/− mice. *n* corresponds to the total number of mice, and the percentages (%) indicate the number of mice with the indicated pathology

	p53−/− AMPK β1+/+		p53−/− AMPK β1−/−	
Sample size	19 (17 males, 2 females)		20 (17 males, 3 females)	
Incidence	*n*	%	*n*	%
Tumors				
Lymphoma	16	84	17	85
Soft tissue tumors	5	26	3	15
Lymphoid leukemia			1	5

### AMPK β1 deficiency reduces ACC phosphorylation and accelerates lipogenesis

**Figure 3:**
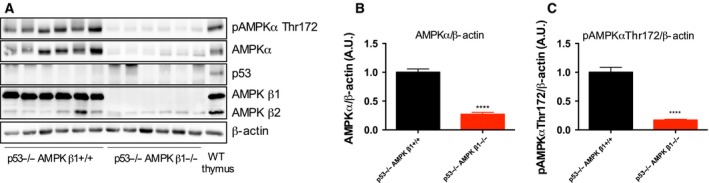
Impaired AMPK phosphorylation in tumors harvested from the p53−/− AMPK β1−/− mice. (A) Tumors harvested from p53−/− AMPK β1+/+ and p53−/− AMPK β1−/− mice were homogenized and subjected to immunoblotting using the indicated antibodies. Six animals per genotype are shown. A thymus from a WT mouse was used as a control. Bar graphs show densitometry of (B) AMPKα/β‐actin, C) pAMPKα Thr172/β‐actin. Results are the means ± SEM. *P < 0.05, **P < 0.01, ***P < 0.001, ****P < 0.0001 using two‐tailed Student's *t*‐test.

We performed immunoblot analysis of tumor protein extracts collected at endpoint and found that p53−/− AMPK β1−/− tumors had large reductions in total AMPK α expression and AMPK activating phosphorylation at Thr172 (Fig. 3A–C). As anticipated, there was no detectable p53, and importantly, there appeared to be no compensatory increase in AMPK β2 expression (Fig. 3A). These data indicate that tumors of p53−/− AMPK β1−/− mice had large reductions in AMPK activating phosphorylation.

AMPK may regulate tumorigenesis through multiple mechanisms so we examined phosphorylation of key downstream substrates. One way AMPK may inhibit tumorigenesis is through phosphorylation of ULK1 at Ser555 and subsequent upregulation of autophagy (Mihaylova and Shaw, 2011). There was a tendency for a reduced ratio of pULK1 S555 to ULK1 in tumors of p53−/− AMPK β1−/− mice (Fig. S2A,B). However, there were no other signs of dysregulated autophagy as inhibitory phosphorylation of ULK1 S757/ULK1 (Fig. S2A,C), the expression of p62 (Fig. S2A,D) and ATG13 (Fig. S2A,E), and the ratio of LC3BII to LC3BI (Fig. S2F,G) were all comparable between genotypes. AMPK may also limit tumorigenesis by inhibiting mTOR activity (Mihaylova and Shaw, 2011), but we found that three downstream markers of mTOR activity (S6K1 and S6 kinases and HIF1α) were similar between genotypes (Fig. S3A–C). Activating phosphorylation of Akt was also unaltered (Fig. S3A,D–E). As mitochondrial content is regulated by AMPK (O'Neill *et al*., 2013) and has emerged as an important factor regulating tumor progression (Hirschey *et al*., 2015), we measured components of the electron transport chain and found that they were also comparable between p53−/− AMPK β1+/+ and p53−/− AMPK β1−/− mice, suggesting that tumor mitochondrial content was not altered (Fig. S3A,F). These data suggest reductions in autophagy and mitochondrial content or increases in Akt and mTOR did not contribute to the increased tumor progression in p53−/− AMPK β1−/− mice.

As AMPK β1 is important for inhibiting inflammation (Galic *et al*., 2011) and inflammation can accelerate lymphoma development (Grivennikov *et al*., 2011), we measured the mRNA and protein expression of inflammatory cytokines. We found that the mRNA and protein expression of inflammatory markers were not altered with the exception of IL1β protein which was elevated in tumors of p53−/− AMPK β1−/− mice (Table S1). However, this increase in IL1β was not accompanied by elevated activating phosphorylation of c‐Jun N‐terminal kinase (JNK), which is known to be downstream of the IL1 receptor (Fig. S3A,G). There was also no difference in the activating phosphorylation (S727 or Y705) of the signal transducer and activator of transcription 3 (STAT3) (Fig. S3A,H,I).

**Figure 4:**
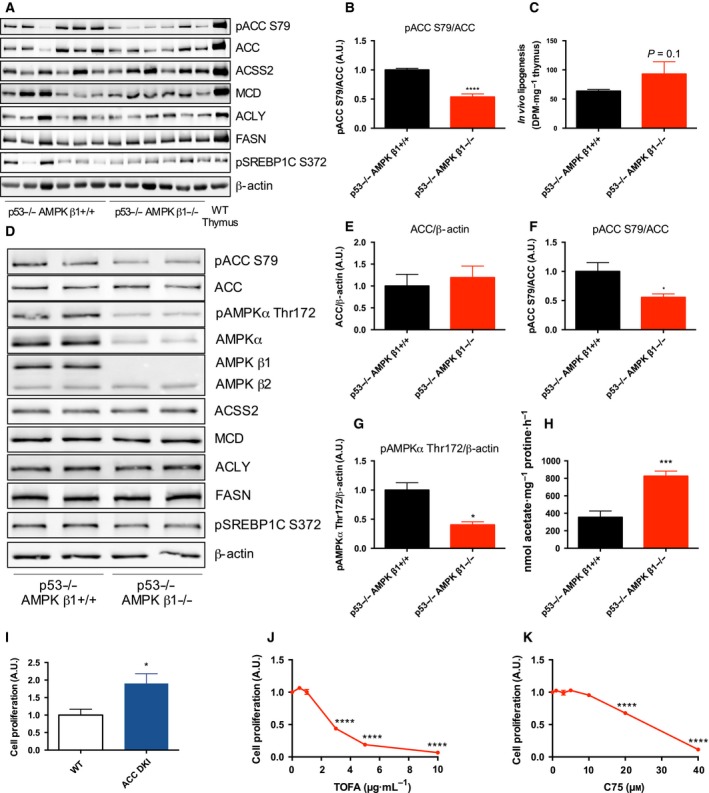
AMPK β1 deficiency accelerates lipogenesis. (A) Tumors harvested from p53−/− AMPK β1+/+ and p53−/− AMPK β1−/− mice were homogenized and subjected to immunoblotting using the indicated antibodies. Six animals per genotype are shown. A thymus from a WT mouse was used as a control. Bar graphs show densitometry of (B) pACC Ser79/ACC immunoblots. (C) *In vivo* lipogenesis in p53−/− AMPK β1+/+ and p53−/− AMPK β1−/− mice. *n* = 5–8 mice per genotype. (D) p53−/− AMPK β1+/+ and p53−/− AMPK β1−/− MEFs were subjected to immunoblotting with the indicated antibodies. Bar graphs show densitometry of (E) ACC/β‐actin, (F) pACC Ser79/ACC, and (G) pAMPKα Thr172/β‐actin immunoblots. *n* = 2–3 independent experiments in duplicate. (H) Lipogenesis in p53−/− AMPK β1+/+ and p53−/− AMPK β1−/− MEFs. *n* = 3 independent experiments in triplicate. (I) Cell proliferation was measured in p53+/+ AMPK β1+/+ and ACC DKI MEFs. *n* = 4 independent experiments. p53−/− AMPK β1−/− MEFs were treated with increasing concentrations of (J) TOFA and (K) C75, and cell proliferation was then measured. *n* = 2–3 independent experiments. Results are the means ± SEM. *P < 0.05, **P < 0.01, ***P < 0.001, ****P < 0.0001 using two‐tailed Student's *t*‐test (for A–I) and one‐way ANOVA and Fisher's post hoc test (for J,K).

Elevated rates of lipogenesis are used to fuel membrane biosynthesis in rapidly proliferating cells (Menendez and Lupu, 2007; Zadra *et al*., 2014). ACC is an important enzyme regulating lipogenesis and is inhibited by AMPK following phosphorylation at Ser79 (Fullerton *et al*., 2013; Munday *et al*., 1988). We found that consistent with reductions in AMPK activating phosphorylation, there was a reduction of approximately 50% in phosphorylation of ACC at Ser79 compared to the p53−/− AMPK β1+/+ tumors (Fig. 4A,B). There were no alterations in the expression of other lipogenic enzymes [acetyl‐CoA synthetase (ACCSS2), malonyl‐CoA decarboxylase (MCD), ATP‐citrate lyase (ACLY), fatty acid synthase (FASN), and phosphorylation of S372 on sterol regulatory element‐binding protein 1c (SREBP1c)]. Consistent with reductions in ACC phosphorylation, we measured *in vivo* lipogenesis in 3‐month‐old mice and found that p53−/− AMPK β1−/− mice tended to have an increased incorporation of acetate into fatty acids compared to p53−/− AMPK β1+/+ mice (Fig. 4C). These data indicate that genetic deletion of AMPK β1 leads to reductions in ACC phosphorylation and tends to increase *de novo* lipogenesis *in vivo*.

To further examine the mechanisms by which lipogenesis may regulate cellular proliferation, we generated mouse embryonic fibroblasts (MEFs) from p53−/− AMPK β1+/+ and p53−/− AMPK β1−/− littermates. Consistent with immunoblotting in tumors, we found that p53−/− AMPK β1−/− MEFs (Fig. 4D) had reductions in AMPK and ACC phosphorylation (Fig. 4D–G), but not alterations in the expression of other regulators of lipogenesis (ACSS2, MCD, ACLY, FASN, pSREBP1c, Fig. 4D). Similar to observations in the tumor, this reduction in ACC phosphorylation was associated with a significant increase in lipogenesis (Fig. 4H). These data suggest that reduced AMPK‐mediated phosphorylation of ACC was important for increasing lipogenesis and the subsequent proliferative capacity of p53−/− AMPK β1−/− mice via a cell autonomous pathway.

To directly evaluate the importance of AMPK phosphorylation of ACC in regulating cellular proliferation, we examined MEFs derived from mice with Ser‐Ala‐knock‐in mutations in the AMPK phosphorylation site on ACC1 (S79A) and ACC2 (S212A) (ACC DKI), which makes ACC constitutively active and increases rates of lipogenesis (Fullerton *et al*., 2013). We found that MEFs derived from ACC DKI mice had increased proliferation rates by 1.9‐fold compared to WT controls (Fig. 4I), indicating that AMPK regulation of ACC phosphorylation is vital for limiting cellular proliferative capacity. To further examine the importance of lipogenesis in regulating cellular proliferation, we treated MEFs with TOFA and C75, two inhibitors of lipogenesis that act downstream of AMPK β1 to inhibit ACC and fatty acid synthase activity, respectively, and found that both agents dose dependently inhibited cellular proliferation (Fig. 4J,K). Collectively, these data suggest that AMPK β1 inhibition of lipogenesis, through phosphorylation of ACC, may be important for limiting proliferation in the absence of p53.

## Discussion

Reductions in AMPK activity can occur under a number of pathological conditions known to increase cancer incidence. These include inactivating mutations in the tumor suppressors p53 and LKB1 or elevated levels of glucose (Friedrichsen *et al*., 2013), insulin (Ruderman *et al*., 2013), TNF (Steinberg *et al*., 2006), and LPS (Galic *et al*., 2011). In contrast, elevated levels of AMPK β1 have been associated with increased survival in lymphoma and ovarian cancer (Hoffman *et al*., 2013; Li *et al*., 2012). In the current study, we provide the first genetic evidence that deletion of the AMPK β1 isoform accelerates the progression of a T‐cell lymphoma that reduces the survival of mice that are heterozygous or homozygous null for p53. Interestingly, the deletion of AMPK β1 did not alter the spectrum of cancer development compared to p53 deficiency alone, in that both mice still developed T‐cell lymphoma. This may be because AMPK β1 is the predominant subunit expressed in the thymus and hematopoietic tissues and hence these tissues, which are also the most sensitive to the loss of p53, also had the greatest reduction in AMPK activity. While our findings may only be applicable to T‐cell lymphoma, it is intriguing to speculate that because p53 loss‐of‐function mutations are associated with approximately 80% of all cancers, these findings may be applicable to other types of cancer where the AMPK β1 subunit predominates, such as the liver, lung, gastrointestinal tract, or ovaries.

Previous studies by Faubert *et al*. (2013) found that genetic deletion of AMPK α1 accelerates Myc‐driven lymphoma due to elevations in mTORC1 activity and subsequent increases in HIF1α that promoted aerobic glycolysis (Warburg effect). In contrast to these findings, we found that AMPK β1‐p53 null tumors did not have altered activity of mTORC1 (as detected by normal phosphorylation of downstream substrates S6K, S6, and ULK1 S757) or HIF1α. We also detected no change in markers of autophagy or mitochondrial content. Instead, we found that AMPK β1 p53 null tumors and MEFs had increased rates of lipogenesis that was not associated with alterations in the expression of fatty acid synthetic enzymes but instead was directly related to reduced phosphorylation of ACC. Proliferation data from the ACC DKI MEFs or cells treated with lipogenesis inhibitors corroborated these results, suggesting that AMPK control of lipogenesis, through ACC inhibition, is important for limiting cellular proliferation. These data are in agreement with previous studies indicating that hepatocytes (Dzamko *et al*., 2010) and macrophages (Fullerton *et al*., 2015) from AMPK β1‐deficient mice have increased lipid synthesis and that activation of AMPK using A769662 or metformin lowers lipogenesis through an AMPK β1‐dependent pathway requiring the phosphorylation of ACC (Fullerton *et al*., 2013). These data are also consistent with recent studies indicating that inhibiting lipogenesis, by mimicking AMPK phosphorylation of ACC using small molecules (Svensson *et al*., 2016) or through chemical inhibition of fatty acid synthase (Menendez and Lupu, 2007; Orita *et al*., 2008), is effective for limiting cellular proliferation. However, it is possible that the inhibition of proliferation is not directly related to the blunting of lipid synthesis but rather a buildup of malonyl‐CoA or some other metabolite. Furthermore, under conditions of metabolic stress that are common within the tumor microenvironment, it is possible that increases in lipid synthesis may be toxic for tumor survival as previously described (Jeon *et al*., 2012). Future studies in mice lacking p53 and ACC1 S79 and ACC2 S212 phosphorylation are required to directly establish the importance of this lipogenic pathway in limiting proliferation and survival.

In addition to increases in lipogenesis, we also detected elevated IL1β protein (but not other inflammatory proteins) in AMPK β1p53 null tumors. In contrast to other inflammatory cytokines (e.g., TNFa, IL6), increases in IL1β protein are the result of the induction of the NLRP3 (NOD‐like receptor family, pyrin domain containing) inflammasome pathway (Haneklaus *et al*., 2013; Steinberg and Schertzer, 2014). Production of bioactive IL1β by the NLRP3 inflammasome requires a two‐step process involving initial priming from the toll‐like receptors (TLR) by lipopolysaccharide (LPS) and subsequent activation by hyperglycemia, saturated fatty acids such as palmitate, ROS, oxidized LDL, or cholesterol crystals. This leads to NLRP3 assembly with a protein complex containing caspase‐1 and consequent processing and secretion of mature IL1β (Haneklaus *et al*., 2013; Steinberg and Schertzer, 2014). Importantly, the NLRP3 inflammasome can be inhibited by AMPK (Wen *et al*., 2011), although the mechanisms are not fully understood [for review, see (Steinberg and Schertzer, 2014)]. So while the role of IL1β in tumorigenesis is context dependent and is largely dependent on the tumor microenvironment, it is possible that AMPK inhibition of the NLRP3 inflammasome may also be important for reducing tumorigenesis in p53 null mice. However, future studies are required to directly investigate this concept.

Our study had several limitations. First, we did not examine the morphology of tumors before endpoint. Earlier analysis of tumors could have potentially led to detectable differences in signaling pathways, histology, and tumor sizes between genotypes. Consistent with this idea, there were strong tendencies for increases in both FDG uptake and lipogenesis in p53−/− AMPK β1−/− mice at 12 weeks of age, which was 6–10 weeks before endpoint. Another limitation of our study was that we did not examine the effects of AMPK β1 deletion specifically in T cells. This means that increases in tumorigenesis and reduced survival in p53−/− AMPK β1−/− could have been due to other factors besides the primary lesion. For example, AMPK α1, β1, and γ1 null mice have been shown to have increased red blood cell fragility, which causes microcytic anemia and splenomegaly without altering the number of leukocytes (Cambridge *et al*., 2017; Föller *et al*., 2009; Foretz *et al*., 2010, 2011). We also observed splenomegaly in our AMPK β1 null mice (Table S2) so it is possible that this could have an impact on tumorigenesis and lifespan of p53−/− AMPK β1−/− mice. Future studies investigating deletion of AMPK β1 and p53 specifically in T cells will be important for establishing the exact tissues contributing to the accelerated tumorigenesis and reduced longevity of p53−/− AMPK β1−/− mice.

In conclusion, our studies provide the first *in vivo* evidence for a role of the AMPK β1 isoform in limiting the progression of tumorigenesis that results from a deficiency in p53. Increased tumorigenesis in this model was associated with reduced AMPK phosphorylation of ACC and elevated IL1β and lipogenesis, indicating that the maintenance of AMPKβ1 expression is important for limiting the progression of p53‐deficient tumors.

## Materials and methods

### Materials

Complete protease inhibitor cocktail tablets were obtained from Roche (Laval, QC, Canada). [^3^H]Acetic acid was from PerkinElmer (Waltham, MA, USA). Cell culture media and reagents were from Life Technologies (Burlington, ON, Canada). TOFA (5‐(tetradecyloxy)‐2‐furoic acid) and C75 ((2R*,3S*)‐tetrahydro‐4‐methylene‐2‐octyl‐5‐oxo‐3‐furancarboxylic acid) were purchased from Cayman Chemical (Ann Arbor, MI, USA). All other chemicals were purchased from Sigma (St. Louis, MO, USA).

### Antibodies

The anti‐phospho‐ACC S79 (catalogue # 3661), anti‐ACC (catalogue # 3676), anti‐phospho‐AMPKα Thr172 (catalogue # 2535), anti‐AMPKα (catalogue # 2532), anti‐AMPK β1/β2 (catalogue # 4150), anti‐p53 (catalogue # 2524), anti‐β‐actin (catalogue # 5125), anti‐acetyl‐CoA synthetase (ACSS2) (catalogue # 3658), anti‐pULK1 S555 (catalogue # 5569), anti‐phospho‐ULK1 S757 (catalogue # 6888), anti‐ULK1 (catalogue # 8054), anti‐p62 (catalogue # 5114), anti‐ATG13 (catalogue # 6940), anti‐LC3B (catalogue # 3868), anti‐phospho‐S6K1 Thr389 (catalogue # 9205), anti‐S6K1 (catalogue # 9202), anti‐phospho‐S6 S240/244 (catalogue # 5364), anti‐S6 (catalogue # 2217), anti‐phospho‐Akt S473 (catalogue # 4058), anti‐phospho‐Akt Thr308 (catalogue # 4056), anti‐Akt (catalogue # 9272), anti‐ATP‐citrate lyase (ACLY) (catalogue # 4332), anti‐fatty acid synthase (Fasn) (catalogue # 3189), anti‐phospho‐sterol regulatory element‐binding protein 1c‐S372 (SREBP1c) (catalogue # 9874), anti‐HIF1α (catalogue # 3716), anti‐phospho‐c‐Jun N‐terminal kinase Thr183/Y185 (JNK) (catalogue # 4668), anti‐phospho‐signal transducer and activator of transcription 3 S727 (STAT3) (catalogue # 9134), and anti‐phospho‐STAT3 Y705 (catalogue # 9145) were purchased from Cell Signaling Technology (Danvers, MA, USA). Malonyl‐CoA decarboxylase (MCD) (catalogue # ab95945) antibody was purchased from Abcam (Cambridge, MA, USA). The anti‐CD3 (MRQ‐39) antibody used for immunohistochemistry was from CELL MARQUE (Rocklin, CA, USA). The anti‐OXPHOS antibody was purchased from MitoSciences (Eugene, OR, USA). The anti‐secondary antibodies coupled to HRP (horseradish peroxidase) were from Cell Signaling Technology.

### Generation of p53−/− AMPK β1−/− mice

All experiments were approved by the McMaster University Animal Ethics Committee and conducted under the Canadian guidelines for animal research. Animals were housed in microisolator cages located in a room maintained at 23 **°**C with a 12‐h light/dark cycle and fed with normal chow diet *ad libitum*. p53+/− mice on a C57BL/6 background were purchased from The Jackson Laboratory (B6.129S2‐Trp53^tm1Tyj/J^, stock 002101; Bar Harbor, ME, USA). AMPK β1 null mice (Dzamko *et al*., 2010) were generated on a C57Bl6 background as described and p53 null mice had been backcrossed to a C57Bl6 background for at least 10 generations. Heterozygous mice (p53+/−) were interbred to generate p53−/− and p53+/+ littermates. The p53−/− mice were then crossed with the AMPK β1−/− mice (Dzamko *et al*., 2010) to generate the p53+/− AMPK β1+/− mice. These mice were then interbred to generate the p53−/− AMPK β1−/− mice on a C57Bl6 background. Lifespan was determined in a blinded manner by an animal technician who monitored whether mice were found dead or whether mice had reached endpoint according to established parameters [weight loss (>20%), hunched posture, lethargy, labored breathing]. Routine pathology analysis of formalin‐fixed tissues harvested from mice culled at endpoint was performed at the Department of Pathobiology, Ontario Veterinary College, University of Guelph (Ontario, Canada).

### Generation of MEFs

p53−/− AMPK β1+/+, p53−/− AMPK β1−/−, and ACC DKI (S79A in ACC1 and S212A in ACC2) MEFs isolated from mouse embryos at E13.5 (embryonic day 13.5) were generated as described previously (Houde *et al*., 2014) and immortalized by continuous passaging. Cells were cultured in DMEM (25 mm glucose) containing 10% FBS, 4 mm L‐glutamine, 1 mm sodium pyruvate, 1X antibiotic/antimycotic solution, and 1X nonessential amino acids solution.

### Mouse imaging

[^18^F]Fluorodeoxyglucose (FDG) was synthesized at McMaster University and injected into three‐month‐old male mice as previously described (Palanivel *et al*., 2012). FDG uptake was quantified as described (Crane *et al*., 2015).

### Cell proliferation

5 × 10^2^ cells per well were seeded on 96‐well plate and grown for 5 days before being fixed with 10% formalin, stained with 0.5% crystal violet, washed with H_2_O, and dried at 37 **°**C. The crystal violet was then solubilized with 0.05 M NaH_2_PO_4_ and the absorbance measured at 570 nm as described (O'Brien *et al*., 2015). Proliferation rate was compared from untreated p53+/+ AMPK β1+/+ and ACC DKI MEFs. p53−/− AMPK β1−/− MEFs were also treated with TOFA and C75 at the indicated concentrations 24 h after seeding, and proliferation was monitored as described above.

### Immunoblotting

Cells were washed with PBS and lysed in ice‐cold lysis buffer (20 mm Tris/HCl, 150 mm NaCl, 1 mm EDTA, 1 mm EGTA, 1% Triton X‐100, 2.5 mm Na_4_O_7_P_2_, 1 mm Na_3_VO_4_, and Roche inhibitor cocktail). Tissues were homogenized on ice using a Polytron in a 10‐fold mass excess of ice‐cold lysis buffer. Lysates were clarified by centrifugation at 19 000 ***g*** for 15 min at 4 **°**C. Protein concentrations were measured with a BCA assay. Tissue or cell lysates (10–20 μg) were heated in SDS sample buffer and subjected to SDS/PAGE followed by electrotransfer on to nitrocellulose membranes. Membranes were blocked in TBST [50 mm Tris/HCl (pH 7.5), 0.15 mm NaCl, and 0.1% Tween] containing 5% BSA for 1 h. The membranes were then probed with the primary antibody (1000‐fold dilution) for 16 h at 4 **°**C in TBST 5% BSA. Detection of protein was performed using HRP‐conjugated secondary antibodies and an ECL reagent. Immunoblots were analyzed using imagej software (NIH, Bethesda, MD, USA).

### Measurement of lipogenesis

*In vitro,* cells were labeled for 4 h with 0.5 mm sodium acetate and 10 μCi mL^−1^ [^3^H]acetic acid in growth media. Cells were washed three times with ice‐cold PBS and then scraped into chilled PBS. Cells were pelleted by centrifugation at 2000 ***g*** for 2 min. A methanol/chloroform lipid extraction was performed on the cell pellets. After the extraction, the insoluble material (pellet) was dissolved in 0.5 m NaOH/0.5% SDS in order to quantify the amount of protein per well. The incorporation of [^3^H] into the lipid was measured by scintillation counting. The incorporation rate was defined in units of nmol of acetate/mg of protein/h.

*In vivo*, 12‐week‐old mice were fasted for 16 h and refed for 2 h. 20 μCi of [^3^H]acetic acid was then injected intraperitoneally (IP) for 1 h. *De novo* lipogenesis was determined *in vivo* by the incorporation of [^3^H]acetate into thymic lipids.

### Immunohistochemistry lymphoma

Formalin‐fixed and paraffin‐embedded 5‐μm sections were stained with hematoxylin and eosin or an anti‐CD3 antibody (Pearse, 2006), using BENCHMARK ULTRA VENTANA accordingly to the manufacturer's instructions (Roche).

### mRNA expression

For mRNA analysis, tissues were lysed in TRIzol reagent (Life Technologies) and RNA was extracted for qRT‐PCR and mRNA assessed as described (Galic *et al*., 2011).

### Enzyme‐linked immunosorbent assay

Tumors (50–100 mg) were homogenized on ice using a Polytron in ice‐cold lysis buffer 2 (catalogue # 895347, R&D Systems, Minneapolis, MN, USA) containing Roche inhibitor cocktail. Lysates were clarified by centrifugation at 19 000****** ***g*** for 15 min at 4 **°**C. Protein concentrations were measured with a BCA assay. 500 μg of protein lysate was then used to measure IFNγ (Quantikine, R&D Systems), IL6, IL1β, and TNFα (DuoSet, R&D Systems) according to the manufacturer's instructions.

### Statistical analysis

Results are expressed as mean ± SEM. Statistical analyses were performed using two‐tailed Student's *t*‐test or one‐way ANOVA when appropriate. Fisher's least significant difference was used. Significance was set at *P <* *0.05 using graphpad prism 6 software (La Jolla, CA, USA).

## Author contributions

VPH and GRS designed research studies, analyzed data, and wrote the manuscript. VPH, SD, AS, SG, JAH, RAF, GB, and GG conducted experiments and analyzed data. VPH, GRS, SD, AS, SG, JLB, RAF, TT, BEK, GB, and PM edited the manuscript.

## Supporting information


**Fig. S1.** Breeding strategy. Breeding strategy employed to generate the p53−/− AMPK β1−/− mice is shown with the percentage and number of mice for each genotype obtained indicated.

**Fig. S2.** Tumors isolated from p53−/− AMPK β1−/− mice do not have signs of impaired autophagy.

**Fig S3.** Tumors isolated from p53−/− AMPK β1−/− mice do not show alterations in several pathways implicated in accelerated tumorigenesis.

**Table S1.** Relative mRNA expression and protein (pg/mg protein) were measured from tumors isolated from p53−/− AMPK β1+/+ and p53−/− AMPK β1−/− mice collected at endpoint.
**Table S2.** Spleen weight in p53+/+ AMPK β1+/+, p53+/+ AMPK β1−/−, p53−/− AMPK β1+/+ and p53−/− AMPK β1−/− mice. *n* corresponds to the total number of mice.

## References

[mol212079-bib-0001] Adamovich Y , Adler J , Meltser V , Reuven N and Shaul Y (2014) AMPK couples p73 with p53 in cell fate decision. Cell Death Differ 21, 1451–1459. 24874608 10.1038/cdd.2014.60 PMC4131177

[mol212079-bib-0002] Blagih J , Coulombe F , Vincent EE , Dupuy F , Galicia‐Vázquez G , Yurchenko E , Raissi TC , van der Windt GJW , Viollet B , Pearce EL *et al* (2015) The energy sensor AMPK regulates T cell metabolic adaptation and effector responses in vivo. Immunity 42, 41–54. 25607458 10.1016/j.immuni.2014.12.030

[mol212079-bib-0003] Cambridge EL , McIntyre Z , Clare S , Arends MJ , Goulding D , Isherwood C , Caetano SS , Ballesteros Reviriego C , Swiatkowska A , Kane L *et al* (2017) The AMP‐activated protein kinase beta 1 subunit modulates erythrocyte integrity. Exp Hematol 45, 64–68.e5. 27666489 10.1016/j.exphem.2016.09.006 PMC5823972

[mol212079-bib-0004] Carling D , Thornton C , Woods A and Sanders MJ (2012) AMP‐activated protein kinase: new regulation, new roles? Biochem J 445, 11–27. 22702974 10.1042/BJ20120546

[mol212079-bib-0005] Cool B , Zinker B , Chiou W , Kifle L , Cao N , Perham M , Dickinson R , Adler A , Gagne G , Iyengar R *et al* (2006) Identification and characterization of a small molecule AMPK activator that treats key components of type 2 diabetes and the metabolic syndrome. Cell Metab 3, 403–416. 16753576 10.1016/j.cmet.2006.05.005

[mol212079-bib-0006] Crane JD , Palanivel R , Mottillo EP , Bujak AL , Wang H , Ford RJ , Collins A , Blümer RM , Fullerton MD , Yabut JM *et al* (2015) Inhibiting peripheral serotonin synthesis reduces obesity and metabolic dysfunction by promoting brown adipose tissue thermogenesis. Nat Med 21, 166–172. 25485911 10.1038/nm.3766 PMC5647161

[mol212079-bib-0007] Donehower LA , Harvey M , Slagle BL , McArthur MJ , Charles AMJ , Butel JS and Bradley A (1992) Mice deficient for p53 are developmentally normal but susceptible to spontaneous tumours. Nature 356, 215–221. 1552940 10.1038/356215a0

[mol212079-bib-0008] Dzamko N , van Denderen BJW , Hevener AL , Jørgensen SB , Honeyman J , Galic S , Chen Z‐P , Watt MJ , Campbell DJ , Steinberg GR *et al* (2010) AMPK β1 deletion reduces appetite, preventing obesity and hepatic insulin resistance. J Biol Chem 285, 115–122. 19892703 10.1074/jbc.M109.056762 PMC2804155

[mol212079-bib-0009] Faubert B , Boily G , Izreig S , Griss T , Samborska B , Dong Z , Dupuy F , Chambers C , Fuerth BJ , Viollet B *et al* (2013) AMPK is a negative regulator of the Warburg effect and suppresses tumor growth in vivo. Cell Metab 17, 113–124. 23274086 10.1016/j.cmet.2012.12.001 PMC3545102

[mol212079-bib-0010] Faubert B , Vincent EE , Poffenberger MC and Jones RG (2015) The AMP‐activated protein kinase (AMPK) and cancer: many faces of a metabolic regulator. Cancer Lett 356, 165–170. 24486219 10.1016/j.canlet.2014.01.018

[mol212079-bib-0011] Föller M , Sopjani M , Koka S , Gu S , Mahmud H , Wang K , Floride E , Schleicher E , Schulz E , Münzel T *et al* (2009) Regulation of erythrocyte survival by AMP‐activated protein kinase. FASEB J 23, 1072–1080. 19050047 10.1096/fj.08-121772

[mol212079-bib-0012] Forbes SA , Beare D , Gunasekaran P , Leung K , Bindal N , Boutselakis H , Ding M , Bamford S , Cole C , Ward S *et al* (2015) COSMIC: Exploring the world's knowledge of somatic mutations in human cancer. Nucleic Acids Res 43, D805–D811. 25355519 10.1093/nar/gku1075 PMC4383913

[mol212079-bib-0013] Foretz M , Guihard S , Leclerc J , Fauveau V , Couty JP , Andris F , Gaudry M , Andreelli F , Vaulont S and Viollet B (2010) Maintenance of red blood cell integrity by AMP‐activated protein kinase alpha1 catalytic subunit. FEBS Lett 584, 3667–3671. 20670625 10.1016/j.febslet.2010.07.041

[mol212079-bib-0014] Foretz M , Hébrard S , Guihard S , Leclerc J , Do Cruzeiro M , Hamard G , Niedergang F , Gaudry M and Viollet B (2011) The AMPKγ1 subunit plays an essential role in erythrocyte membrane elasticity, and its genetic inactivation induces splenomegaly and anemia. FASEB J 25, 337–347. 20881209 10.1096/fj.10-169383

[mol212079-bib-0015] Friedrichsen M , Mortensen B , Pehmøller C , Birk JB and Wojtaszewski JFP (2013) Exercise‐induced AMPK activity in skeletal muscle: role in glucose uptake and insulin sensitivity. Mol Cell Endocrinol 366, 204–214. 22796442 10.1016/j.mce.2012.06.013

[mol212079-bib-0016] Fullerton MD , Ford RJ , McGregor CP , LeBlond ND , Snider SA , Stypa SA , Day EA , Lhoták Š , Schertzer JD , Austin RC *et al* (2015) Salicylate improves macrophage cholesterol homeostasis via activation of Ampk. J Lipid Res. 56, 1025–1033. 25773887 10.1194/jlr.M058875 PMC4409279

[mol212079-bib-0017] Fullerton MD , Galic S , Marcinko K , Sikkema S , Pulinilkunnil T , Chen Z‐P , O'Neill HM , Ford RJ , Palanivel R , O'Brien M *et al* (2013) Single phosphorylation sites in Acc1 and Acc2 regulate lipid homeostasis and the insulin‐sensitizing effects of metformin. Nat Med 19, 1649–1654. 24185692 10.1038/nm.3372 PMC4965268

[mol212079-bib-0018] Galic S , Fullerton M , Schertzer J , Sikkema S , Marcinko K , Walkley C , Izon D , Honeyman J , Chen Z , van Denderen B *et al* (2011) Hematopoietic AMPK beta1 reduces mouse adipose tissue macrophage inflammation and insulin resistance in obesity. J Clin Invest 121, 4903–4915. 22080866 10.1172/JCI58577 PMC3226000

[mol212079-bib-0019] Grivennikov SI , Greten FR and Karin M (2011) Immunity, inflammation, and cancer. Cell 140, 883–899. 10.1016/j.cell.2010.01.025 PMC2866629 20303878

[mol212079-bib-0020] Hanahan D and Weinberg RA (2011) Hallmarks of cancer: the next generation. Cell 144, 646–674. 21376230 10.1016/j.cell.2011.02.013

[mol212079-bib-0021] Haneklaus M , O'Neill LA , Coll RC (2013) Modulatory mechanisms controlling the NLRP3 inflammasome in inflammation: recent developments. Curr Opin Immunol 25, 40–45. 23305783 10.1016/j.coi.2012.12.004

[mol212079-bib-0022] Hawley SA , Fullerton MD , Ross FA , Schertzer JD , Chevtzoff C , Walker KJ , Peggie MW , Zibrova D , Green KA , Mustard KJ *et al* (2012) The ancient drug salicylate directly activates AMP‐activated protein kinase. Science 336, 918–922. 22517326 10.1126/science.1215327 PMC3399766

[mol212079-bib-0023] He G , Zhang Y‐W , Lee J‐H , Zeng SX , Wang YV , Luo Z , Dong XC , Viollet B , Wahl GM and Lu H (2014) AMP‐activated protein kinase induces p53 by phosphorylating MDMX and inhibiting its activity. Mol Cell Biol 34, 148–157. 24190973 10.1128/MCB.00670-13 PMC3911293

[mol212079-bib-0024] Hirschey MD , Deberardinis RJ , Diehl AME , Drew JE , Frezza C , Green MF , Jones LW , Ko YH , Le A , Lea MA *et al* (2015) Dysregulated metabolism contributes to oncogenesis. Semin Cancer Biol 35, S129–S150. 26454069 10.1016/j.semcancer.2015.10.002 PMC4656121

[mol212079-bib-0025] Hoffman AE , Demanelis K , Fu A , Zheng T , Zhu Y (2013) Association of AMP‐activated protein kinase with risk and progression of non‐hodgkin lymphoma. Cancer Epidemiol Biomarkers Prev. 22, 736–744. 23396962 10.1158/1055-9965.EPI-12-1014 PMC3631103

[mol212079-bib-0026] Houde VP , Ritorto MS , Gourlay R , Varghese J , Davies P , Shpiro N , Sakamoto K and Alessi DR (2014) Investigation of LKB1 Ser431 phosphorylation and Cys433 farnesylation using mouse knockin analysis reveals an unexpected role of prenylation in regulating AMPK activity. Biochem J 458, 41–56. 24295069 10.1042/BJ20131324 PMC3898322

[mol212079-bib-0027] Huang X , Wullschleger S , Shpiro N , McGuire VA , Sakamoto K , Woods YL , McBurnie W , Fleming S , Alessi DR (2008) Important role of the LKB1‐AMPK pathway in suppressing tumorigenesis in PTEN‐deficient mice. Biochem J 412, 211–221. 18387000 10.1042/BJ20080557

[mol212079-bib-0028] Jacks T , Remington L , Williams BO , Schmitt EM , Halachmi S , Bronson RT and Weinberg RA (1994) Tumor spectrum analysis in p53‐mutant mice. Curr Biol 4, 1–7. 7922305 10.1016/s0960-9822(00)00002-6

[mol212079-bib-0029] Jeon S‐M , Chandel NS and Hay N (2012) AMPK regulates NADPH homeostasis to promote tumour cell survival during energy stress. Nature 485, 661–665. 22660331 10.1038/nature11066 PMC3607316

[mol212079-bib-0030] Jones RG , Plas DR , Kubek S , Buzzai M , Mu J , Xu Y , Birnbaum MJ and Thompson CB (2005) AMP‐activated protein kinase induces a p53‐dependent metabolic checkpoint. Mol Cell 18, 283–293. 15866171 10.1016/j.molcel.2005.03.027

[mol212079-bib-0031] Lee C‐W , Wong LL‐Y , Tse EY‐T , Liu H‐F , Leong VY‐L , Lee JM‐F , Hardie DG , Ng IO‐L and Ching Y‐P (2012) AMPK promotes p53 acetylation via phosphorylation and inactivation of SIRT1 in liver cancer cells. Cancer Res 72, 4394–4404. 22728651 10.1158/0008-5472.CAN-12-0429 PMC3433393

[mol212079-bib-0032] Li J , Jiang P , Robinson M , Lawrence TS and Sun Y (2003) AMPK‐β1 subunit is a p53‐independent stress responsive protein that inhibits tumor cell growth upon forced expression. Carcinogenesis 24, 827–834. 12771025 10.1093/carcin/bgg032

[mol212079-bib-0033] Li C , Liu VW , Chiu PM , Chan DW and Ngan HY (2012) Over‐expressions of AMPK subunits in ovarian carcinomas with significant clinical implications. BMC Cancer 12, 357. 22897928 10.1186/1471-2407-12-357 PMC3518102

[mol212079-bib-0034] Menendez JA and Lupu R (2007) Fatty acid synthase and the lipogenic phenotype in cancer pathogenesis. Nat Rev Cancer 7, 763–777. 17882277 10.1038/nrc2222

[mol212079-bib-0035] Mihaylova MM and Shaw RJ (2011) The AMPK signalling pathway coordinates cell growth, autophagy and metabolism. Nat Cell Biol 13, 1016–1023. 21892142 10.1038/ncb2329 PMC3249400

[mol212079-bib-0036] Munday MR , Campbell DG , Carling D and Hardie DG (1988) Identification by amino acid sequencing of three major regulatory phosphorylation sites on rat acetyl‐CoA carboxylase. Eur J Biochem 175, 331–338. 2900138 10.1111/j.1432-1033.1988.tb14201.x

[mol212079-bib-0037] O'Brien AJ , Villani LA , Broadfield LA , Houde VP , Galic S , Blandino G , Kemp BE , Tsakiridis T , Muti P and Steinberg GR (2015) Salicylate activates AMPK and synergizes with metformin to reduce the survival of prostate and lung cancer cells ex vivo through inhibition of de novo lipogenesis. Biochem J 469, 177–187. 25940306 10.1042/BJ20150122

[mol212079-bib-0038] O'Neill HM , Holloway GP and Steinberg GR (2013) AMPK regulation of fatty acid metabolism and mitochondrial biogenesis: implications for obesity. Mol Cell Endocrinol 366, 135–151. 22750049 10.1016/j.mce.2012.06.019

[mol212079-bib-0039] Orita H , Coulter J , Tully E , Kuhajda FP and Gabrielson E (2008) Inhibiting fatty acid synthase for chemoprevention of chemically induced lung tumors. Clin Cancer Res 14, 2458–2464. 18413838 10.1158/1078-0432.CCR-07-4177

[mol212079-bib-0040] Palanivel R , Fullerton MD , Galic S , Honeyman J , Hewitt KA , Jorgensen SB and Steinberg GR (2012) Reduced Socs3 expression in adipose tissue protects female mice against obesity‐Induced insulin resistance. Diabetologia 55, 3083–3093. 22872213 10.1007/s00125-012-2665-3 PMC5233443

[mol212079-bib-0041] Pearse G (2006) Toxicologic pathology – normal structure, function and histology of the thymus. Toxicol Pathol 34, 504–514. 17067941 10.1080/01926230600865549

[mol212079-bib-0042] Ross FA , MacKintosh C and Hardie DG (2016) AMP‐activated protein kinase: a cellular energy sensor that comes in twelve flavours. FEBS J 283, 2987–3001. 26934201 10.1111/febs.13698 PMC4995730

[mol212079-bib-0043] Ruderman NB , Carling D , Prentki M and Cacicedo JM (2013) Science in medicine AMPK, insulin resistance, and the metabolic syndrome. J Clin Invest 123, 2764–2772. 23863634 10.1172/JCI67227 PMC3696539

[mol212079-bib-0044] Sanders MJ , Ali ZS , Hegarty BD , Heath R , Snowden MA and Carling D (2007) Defining the mechanism of activation of AMP‐activated protein kinase by the small molecule A‐769662, a member of the thienopyridone family. J Biol Chem 282, 32539–32548. 17728241 10.1074/jbc.M706543200

[mol212079-bib-0045] Scott JW , Ling N , Issa SMA , Dite TA , O'Brien MT , Chen ZP , Galic S , Langendorf CG , Steinberg GR , Kemp BE *et al* (2014) Small molecule drug A‐769662 and AMP synergistically activate naive AMPK independent of upstream kinase signaling. Chem Biol 21, 619–627. 24746562 10.1016/j.chembiol.2014.03.006

[mol212079-bib-0046] Shackelford DB , Abt E , Gerken L , Vasquez DS , Seki A , Leblanc M , Wei L , Fishbein MC , Czernin J , Mischel PS *et al* (2013) LKB1 inactivation dictates therapeutic response of non‐small cell lung cancer to the metabolism drug phenformin. Cancer Cell 23, 143–158. 23352126 10.1016/j.ccr.2012.12.008 PMC3579627

[mol212079-bib-0047] Shah S , Carriveau WJ , Li J , Campbell SL , Piotr K , Lim H , Daurio N , Trefely S and Won K (2016) Targeting ACLY sensitizes castration‐resistant prostate cancer cells to AR antagonism by impinging on an ACLY‐AMPK‐AR feedback mechanism. Oncotarget 7, 43713–43730. 27248322 10.18632/oncotarget.9666 PMC5190055

[mol212079-bib-0048] Steinberg GR and Kemp BE (2009) AMPK in health and disease. Physiol Rev 89, 1025–1078. 19584320 10.1152/physrev.00011.2008

[mol212079-bib-0049] Steinberg GR , Michell BJ , van Denderen BJW , Watt MJ , Carey AL , Fam BC , Andrikopoulos S , Proietto J , Görgün CZ , Carling D *et al* (2006) Tumor necrosis factor alpha‐induced skeletal muscle insulin resistance involves suppression of AMP‐kinase signaling. Cell Metab 4, 465–474. 17141630 10.1016/j.cmet.2006.11.005

[mol212079-bib-0050] Steinberg GR and Schertzer JD (2014) AMPK promotes macrophage fatty acid oxidative metabolism to mitigate inflammation: implications for diabetes and cardiovascular disease. Immunol Cell Biol 92, 1–6. 10.1038/icb.2014.11 24638063

[mol212079-bib-0051] Svensson RU , Parker SJ , Eichner LJ , Kolar MJ , Wallace M , Brun SN , Lombardo PS , Van Nostrand JL , Hutchins A , Vera L *et al* (2016) Inhibition of acetyl‐CoA carboxylase suppresses fatty acid synthesis and tumor growth of non‐small‐cell lung cancer in preclinical models. Nat Med 22, 1108–1119. 27643638 10.1038/nm.4181 PMC5053891

[mol212079-bib-0052] Villani LA , Smith BK , Marcinko K , Ford RJ , Broadfield LA , Green AE , Houde VP , Muti P , Tsakiridis T , Steinberg GR (2016) The diabetes medication Canagliflozin reduces cancer cell proliferation by inhibiting mitochondrial complex‐I supported respiration. Mol Metab 5, 1048–1056. 27689018 10.1016/j.molmet.2016.08.014 PMC5034684

[mol212079-bib-0053] Vincent EE , Coelho PP , Blagih J , Griss T , Viollet B and Jones RG (2015) Differential effects of AMPK agonists on cell growth and metabolism. Oncogene 34, 3627–3639. 25241895 10.1038/onc.2014.301 PMC4980123

[mol212079-bib-0054] Wen H , Gris D , Lei Y , Jha S , Zhang L , Huang MT‐H , Brickey WJ and Ting JP‐Y (2011) Fatty acid‐induced NLRP3‐ASC inflammasome activation interferes with insulin signaling. Nat Immunol 12, 408–415. 21478880 10.1038/ni.2022 PMC4090391

[mol212079-bib-0055] Zadra G , Batista JL and Loda M (2015) Dissecting the dual role of AMPK in cancer: From experimental to human studies. Mol Cancer Res 13, 1059–1172. 25956158 10.1158/1541-7786.MCR-15-0068 PMC4504770

[mol212079-bib-0056] Zadra G , Photopoulos C , Tyekucheva S , Heidari P , Weng QP , Fedele G , Liu H , Scaglia N , Priolo C , Sicinska E *et al* (2014) A novel direct activator of AMPK inhibits prostate cancer growth by blocking lipogenesis. EMBO Mol Med 6, 519–538. 24497570 10.1002/emmm.201302734 PMC3992078

[mol212079-bib-0057] Zhou G , Wang J , Zhao M , Xie T‐X , Tanaka N , Sano D , Patel AA , Ward AM , Sandulache VC , Jasser SA *et al* (2014) Gain‐of‐function mutant p53 promotes cell growth and cancer cell metabolism via inhibition of AMPK activation. Mol Cell 54, 960–974. 24857548 10.1016/j.molcel.2014.04.024 PMC4067806

