# Supplementary material for: AMPK β1 reduces tumor progression and improves survival in p53 null mice
Source: Mol Oncol. 2017 Jun 28;11(9):1143–55. doi: 10.1002/1878-0261.12079 (PMC5579332; doi:10.1002/1878-0261.12079)
Supplement: Supplementary file 1 — Fig. S1. Breeding strategy. Breeding strategy employed to generate the p53−/− AMPK β1−/− mice is shown with the percentage and number of mice for each genotype obtained indicated. [file MOL2-11-1143-s001.pdf]

1) p53+/- x p53+/- → Offspring (Mendelian ratio)

p53+/+	21% (21)
p53+/-	63% (63)
p53-/-	16% (16)

AMPK β1+/- x AMPK β1 +/- → Offspring (Mendelian ratio)

AMPK β1+/+	30% (30)
AMPK β1+/-	41% (41)
AMPK β1-/-	29% (29)

2) AMPK β1-/- x p53-/- → p53+/- AMPK β1+/-

3) p53+/- AMPK β1+/- x p53+/- AMPK β1+/- → Offspring (Mendelian ratio)

p53-/- AMPK β1-/-	2.7% (3)
p53+/+ AMPK β1-/-	5.4% (6)
p53+/- AMPK β1-/-	16% (18)
p53+/+ AMPK β1+/-	11.6% (13)
p53-/- AMPK β1+/-	8.9% (10)
p53+/- AMPK β1+/-	31.3% (35)
p53+/- AMPK β1+/+	13.4% (15)
p53-/- AMPK β1+/+	0.9% (1)
p53+/+ AMPK β1+/+	9.8% (11)

4) p53+/- AMPK β1-/- x p53-/- AMPK β1-/- → Offspring (Mendelian ratio)

p53-/- AMPK β1-/-	44% (7)
p53+/- AMPK β1-/-	56% (9)
