# Supplementary figures and images for: AMPK β1 reduces tumor progression and improves survival in p53 null mice
Source: Mol Oncol. 2017 Jun 28;11(9):1143–55. doi: 10.1002/1878-0261.12079 (PMC5579332; doi:10.1002/1878-0261.12079)

**A**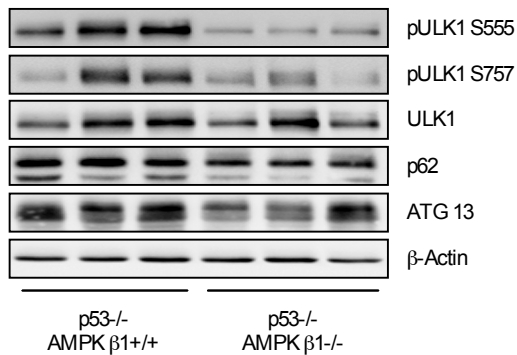**B**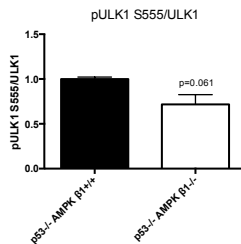**C**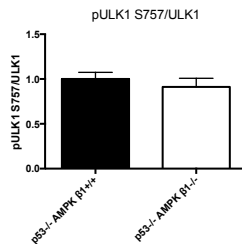**D**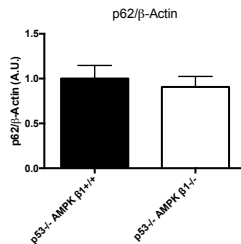**E**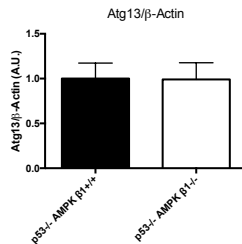**F**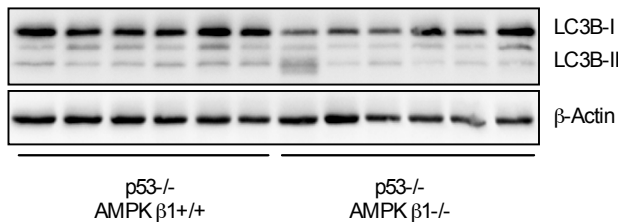**G**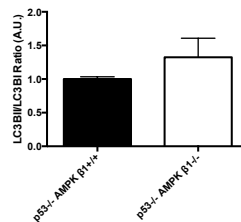

Supplement: Supplementary file 2 — Fig. S2. Tumors isolated from p53−/− AMPK β1−/− mice do not have signs of impaired autophagy. [file MOL2-11-1143-s002.pdf]

**A**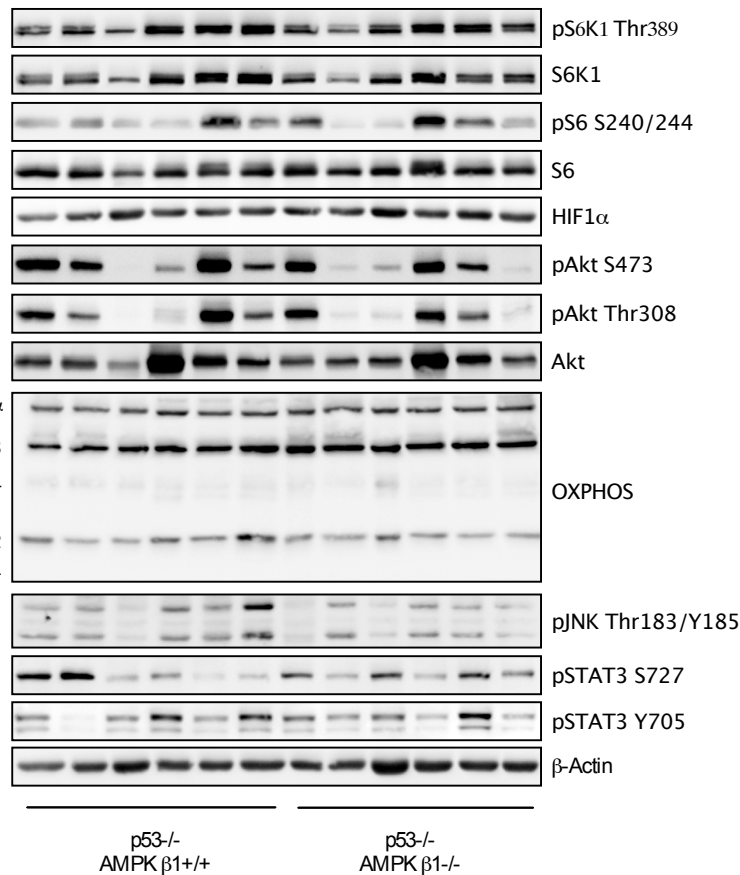**B**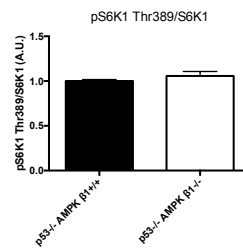**C**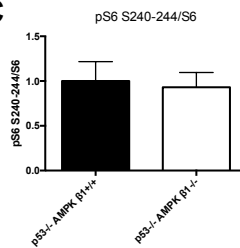**D**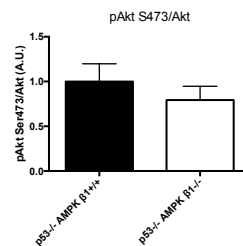**E**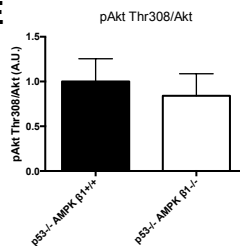**F**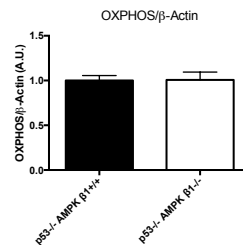**G**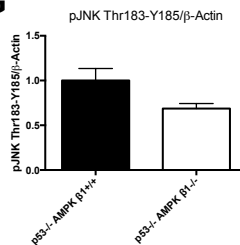**H**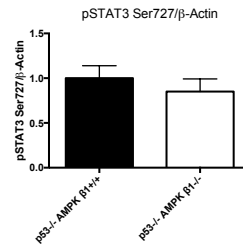**I**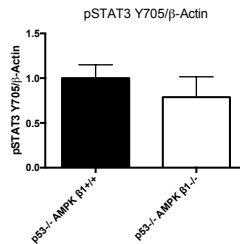

Supplement: Supplementary file 3 — Fig S3. Tumors isolated from p53−/− AMPK β1−/− mice do not show alterations in several pathways implicated in accelerated tumorigenesis. [file MOL2-11-1143-s003.pdf]
