# Supplementary material for: AMPK β1 reduces tumor progression and improves survival in p53 null mice
Source: Mol Oncol. 2017 Jun 28;11(9):1143–55. doi: 10.1002/1878-0261.12079 (PMC5579332; doi:10.1002/1878-0261.12079)
Supplement: Supplementary file 4 — Table S1. Relative mRNA expression and protein (pg/mg protein) were measured from tumors isolated from p53−/− AMPK β1+/+ and p53−/− AMPK β1−/− mice collected at endpoint. Table S2. Spleen weight in p53+/+ AMPK β1+/+, p53+/+ AMPK β1−/−, p53−/− AMPK β1+/+ and p53−/− AMPK β1−/− mice. n corresponds to the total number of mice. [file MOL2-11-1143-s004.docx]

**Supplemental data**

**Supplement Figure Legends**

**Supplementary Figure 1: Breeding strategy.** Breeding strategy employed to generate the p53-/- AMPK β1-/- mice is shown with the percentage and number of mice for each genotype obtained indicated.

**Supplementary Figure 2:** **Tumors isolated from p53-/- AMPK β1-/- mice do not have signs of impaired autophagy. A)** Tumors collected from p53-/- AMPK β1+/+ and p53-/- AMPK β1-/- mice at endpoint were homogenized and subjected to immunoblotting using the indicated antibodies. 3 animals out of 6 per genotype are shown. Bar graphs show densitometry of **B)** pULK1 S555/ULK1 **C)** pULK1 S757/ULK1 **D)** p62/β-Actin and **E)** ATG13/β-Actin immunoblots. **F)** Tumors collected from p53-/- AMPK β1+/+ and p53-/- AMPK β1-/- mice at endpoint were homogenized and subjected to immunoblotting using the LC3B antibody. 6 animals per genotype are shown. **G)** Bar graph shows densitometry of LC3BII/LC3BI Ratio immunoblot. Results are the means ± S.E.M.

**Supplementary Figure 3:** **Tumors isolated from p53-/- AMPK β1-/- mice do not show alterations in several pathways implicated in accelerated tumorigenesis. A)** Tumors collected from p53-/- AMPK β1+/+ and p53-/- AMPK β1-/- mice at endpoint were homogenized and subjected to immunoblotting using the indicated antibodies. 6 animals per genotype are shown. Bar graphs show densitometry of **B)** pS6K1 Thr389/S6K1 **C)** pS6 S240-244/S6 **D)** pAkt S473/Akt **E)** pAkt Thr308/Akt, **F)** OXPHOS/β-Actin **G)** pJNK Thr183-Y185/β-Actin, **H)** pSTAT3 S727/β-Actin and **I)** pSTAT3 Y705/β-Actin immunoblots. Results are the means ± S.E.M.

**Supplemental Tables**

**Supplementary Table 1:** Relative mRNA expression and protein (pg/mg protein) were measured from tumors isolated from p53-/- AMPK β1+/+ and p53-/- AMPK β1-/- mice collected at endpoint.

| **Relative mRNA Expression (A.U.)** | | |
| --- | --- | --- |
| **Gene** | **p53-/- AMPK β1+/+** | **p53-/- AMPK β1-/-** |
| IFNγ | 1.31 ± 0.41 | 2.29 ± 0.89 |
| CD68 | 1.04 ± 0.13 | 1.32 ± 0.37 |
| F4/80 | 1.38 ± 0.35 | 1.30 ± 0.57 |
| IL1β | 1.09 ± 0.21 | 1.66 ± 0.46 |
| IL6 | 1.45 ± 0.42 | 2.20 ± 0.61 |
| TNFα | 1.19 ± 0.32 | 1.36 ± 0.25 |
| iNOS | 1.04 ± 0.31 | 1.94 ± 0.53 |
| Arginase 1 | 1.14 ± 0.28 | 0.85 ± 0.62 |
| **Cytokines (pg/mg protein)** | | |
| **Cytokine** | **p53-/- AMPK β1+/+** | **p53-/- AMPK β1-/-** |
| IL1β | 28.07 ± 6.50 | 55.83 ± 1.99** |
| IFNγ | 4.57 ± 1.8 | 6.79 ± 0.52 |
| TNFα | 19.03 ± 9.65 | 20.73 ± 6.24 |
| IL6 | not detectable | not detectable |

Results are average ± SEM. ** P < 0.01 using two-tailed Student *t* test. n=6-7 animals per group.

**Supplementary Table 2:** Spleen weight in p53+/+ AMPK β1+/+, p53+/+ AMPK β1-/-, p53-/- AMPK β1+/+ and p53-/- AMPK β1-/- mice. n corresponds to the total number of mice.

| **Genotype** | **n** | **Weight (mg)** |
| --- | --- | --- |
| p53+/+ AMPK β1+/+ | 12 | 257.5 ± 14.10 |
| p53+/+ AMPK β1-/- | 15 | 445.73 ± 22.58 **** |
| p53-/- AMPK β1+/+ | 4 | 212.75 ± 80.12 |
| p53-/- AMPK β1-/- | 4 | 325.75 ± 232.43 |

Spleen weights are average ± SEM. **** P < 0.01 using two-tailed Student *t* test.
